# Agreement between the Maslach Burnout Inventory and the Copenhagen Burnout Inventory among emergency physicians and trainees

**DOI:** 10.1111/acem.14994

**Published:** 2024-07-31

**Authors:** Henry Li, Erica Dance, Zafrina Poonja, Leandro Solis Aguilar, Isabelle Colmers‐Gray

**Affiliations:** ^1^ Department of Emergency Medicine, Faculty of Medicine and Dentistry University of Alberta Edmonton Alberta Canada; ^2^ Department of Emergency Medicine, Faculty of Medicine University of British Columbia Vancouver British Columbia Canada; ^3^ Department of Biochemistry University of Alberta Edmonton Alberta Canada; ^4^ Department of Emergency Medicine, School of Medicine Queen's University Kingston Ontario Canada

## Abstract

**Background:**

Emergency physicians have the highest rates of burnout among all specialties. Existing burnout tools include the Copenhagen Burnout Inventory (CBI) and single‐item measures from the Maslach Burnout Inventory (MBI). While both were designed to measure burnout, how they conceptualize this phenomenon differs and their agreement is unclear. Given the close conceptual relationship between emotional regulation strategies such as distancing and distraction with the MBI subscale of depersonalization, we examined agreement between the two inventories and association with emotional regulation strategies as a lens to explore the conceptualization of burnout.

**Methods:**

We conducted a cross‐sectional survey of adult and pediatric emergency physicians and trainees in Canada. Survey questions were pretested using written feedback and cognitive interviews. “Frequent use” of an emotional regulation strategy was “most” or “all” shifts (≥4 on 5‐point Likert scale). Burnout was defined as mean ≥50/100 on the CBI and scoring ≥5 (out of 7) on at least one of the single‐item measures from the MBI. Associations with burnout were examined using multivariable logistic regression.

**Results:**

Of 147 respondents, 44.2% were positive for burnout on the CBI and 44.9% on the single‐item measures from the MBI. Disagreement was 21.1% overall, ranging from 12.5% for older (≥55 years) physicians to 30.2% for younger (<35 years) physicians. Use of distraction and use of distancing were strongly associated with burnout on the single‐item measures (adjusted odds ratio [aOR] 14.4, 95% confidence interval [CI] 3.4–60.8]) and CBI (aOR 10.1, 95% CI 2.5–39.8, respectively.

**Conclusions:**

Despite near‐equal rates of burnout, agreement between the CBI and single‐item measures from the MBI varies and was lower for younger emergency physicians/trainees. While emotional regulation strategies were felt to be important in supporting a career in emergency medicine, they were strongly associated with burnout. Future research is needed to better understand this phenomenon and which tools to use to measure burnout.

## INTRODUCTION

Emergency physicians experience high rates of burnout, depression, and suicidal thoughts.^1^, ^2^ Indeed, over the past decade, emergency medicine has been the specialty with the highest rate of burnout of all medical specialties.^3^, ^4^ In physicians and residents, burnout is associated with an increase in medical errors and worse patient care.^5^, ^6^, ^7^ Furthermore, it can lead to career dissatisfaction, reduced hours, and early retirement,^8^, ^9^, ^10^ costing the United States an estimated $4.6 billion annually.^11^ To advance emergency physician well‐being, we must have a clear understanding of how best to measure burnout.^12^

There are a wide variety of tools that have been developed to measure burnout. The Maslach Burnout Inventory Human Services Survey (MBI)^13^ is by far the most commonly used and studied instrument,^12^ so much so that the construct of burnout itself has often been equated to the three subscales of the MBI (emotional exhaustion, depersonalization, and decreased personal achievement), including by the World Health Organization and the International Classification of Diseases.^14^ Between the three subscales, emotional exhaustion and depersonalization are considered to be the core components of burnout,^12^, ^15^ with the latter sometimes framed as the largest contributor.^12^ Single‐item measures of emotional exhaustion and depersonalization from the MBI have thus seen widespread use in the physician wellness literature, with studies showing strong correlation with the relevant domains of the full MBI^16^ and important associated outcomes.^17^

However, critics of the MBI highlight the conflict of having three separate domains that are each required to fulfill burnout criteria, yet cannot be combined to represent a single unifying construct.^18^, ^19^ Others criticize its narrow focus on the human services sector^20^ as well as ownership by a commercial company,^19^ which has led to the development of other instruments such as the Oldenburg Burnout Inventory and the Copenhagen Burnout Inventory (CBI). Shoman et al.^21^ recently conducted a systematic review of burnout instruments for mental health professionals and found that the CBI appeared the most valid, whereas the MBI had very low quality of evidence for validity.

With varying theoretical conceptualizations of the construct and several inventories that have emerged as candidate measurement instruments, we lack an understanding of how to best measure burnout and the degree of overlap or divergence of these varied conceptualizations.^15^ Our study therefore sought to explore the conceptualization of burnout by examining agreement between the widely used single‐item measures from the MBI as well as the CBI. Recognizing the proposed centrality of depersonalization to the construct of burnout as recognized by the MBI and its absence from the CBI, our study also explored whether emotional regulation strategies such as distancing and distraction (defined in Appendix B, question 12)^22^ which are conceptually similar to depersonalization might be differentially associated with burnout on the two scales.

## METHODS

We conducted a cross‐sectional survey study and have reported results following the CROSS reporting checklist (Appendix A).^23^

### Population and setting

Adult and pediatric emergency medicine physicians and trainees in Canada were eligible to participate. A recent survey of Canadian emergency physician burnout found the prevalence of either high emotional exhaustion or high depersonalization to be 74%,^24^ compared to the general physician population with 53%.^25^ We therefore set the clinically significant difference to be 21%, which requires a sample size of 82 to detect (power = 0.80 and *α* = 0.05).

Emergency departments in Canada are staffed by physicians from a variety of backgrounds. The primary two training routes include a 5‐year specialty residency program (FRCPC) accredited by the Royal College of Physicians and Surgeons of Canada or a 2‐year family medicine residency program followed by a 1‐year enhanced skills program (CCFP‐EM) both accredited by the College of Family Physicians of Canada.^26^ Regional and rural centers are also often staffed by graduates from the 2‐year family medicine residency program. Furthermore, both 5‐year emergency medicine residency program graduates and pediatrics residency program graduates are eligible to complete a 2‐year fellowship in subspecialty pediatric emergency medicine (PEM).^27^ According to Scott's Medical Database, there were 3753 emergency physicians (including family medicine and pediatric emergency medicine) in Canada in 2022.^28^ There are a total of 92 FRCPC residency training spots, 154 CCFP‐EM training spots, and 19 PEM training spots per year.^29^

### Ethics

The study was approved by the University of Alberta Research Ethics Board (Pro00127494). Respondents received study information and implied consent forms before starting the survey. Participants were anonymous at all stages, which prohibited capturing of unique visitors or multiple responses.

### Survey development

We developed a survey (Appendix B) using an approach informed by the Burns methodology,^30^ Gehlbach and Artino's survey checklist,^31^ and Phillips et al.'s reference book for health professions education.^32^ The survey included demographic information, questions on the use of emotional regulation strategies, single‐item measures of emotional exhaustion and depersonalization from the MBI, and the CBI (subscales of personal‐, work‐, and client‐related burnout).

Isbell et al.^22^ recently conducted a qualitative study on emotional regulation in emergency providers and outlined five groups of emotional regulation strategies that we labeled as distancing, distraction, reappraisal, vigilance, and resetting (defined in the survey, Appendix B). We developed questions asking about the frequency of use of the five strategies. Questions were scored on a 5‐point Likert scale ranging from “never”' to “every shift.” The explanations and labels for the categories were reviewed by the aforementioned study's^22^ lead author (LI) to ensure consistency with previous qualitative results. We also included questions on whether respondents were aware of their use of emotional regulation strategies and how important they felt these strategies were to their ability to practice emergency medicine.

We pretested the demographic and emotional regulation questions with eight physicians and residents for comprehensibility, relevance, and comprehensiveness. After adjusting the survey according to this feedback, we conducted cognitive interviews with seven emergency physicians and residents, using the think‐aloud and retrospective probing methods^33^ to examine their thought processes and the content validity of the emotional regulation questions, namely, comprehensiveness, comprehensibility, and relevance. Adjustments were made accordingly and the survey was finalized.

### Survey distribution

The survey was advertised from October to November 2023 via social media (X.com), residency and fellowship program directors, word of mouth, and the Canadian Association of Emergency Physicians (CAEP) survey distribution network. Reminders were sent at 2 and 4 weeks via the CAEP survey network and social media as well as an additional social media reminder at 6 weeks.^34^ All data was collected anonymously using the REDCap platform hosted at the University of Alberta.

### Outcomes

The primary outcome was burnout as determined by the two included burnout scales. The single‐item measures of emotional exhaustion and depersonalization from the MBI are each scored on a 7‐point Likert scale ranging from “never” to “every day.” Burnout was defined as scoring ≥5 (at least “weekly”) on either or both questions^17^ that have been used in both emergency physicians and residents.^35^ The CBI includes 19 questions scored on a 5‐point Likert scale (0, 25, 50, 75, 100). Burnout was defined as a mean of ≥50 on the overall scale.^36^, ^37^ “Frequent use” of each of the five categories of emotional regulation strategies was defined as ≥4 (at least “most shifts”) on a 5‐point Likert scale.

### Data analyses

Data were analyzed using R.^38^ We calculated means with standard deviations (SDs) for continuous variables and proportions with percentages for categorical variables. We calculated the internal consistency of the CBI as well as that of its subscales, with good internal consistency defined as a Cronbach alpha ≥ 0.70.^39^ The agreement of the two definitions of burnout was examined and repeated for subgroups including frequent use of emotional regulation strategies, age and gender categories, and visible minority and trainee status. Univariate associations with agreement were examined using chi‐square analyses with significance set at *p* < 0.05.

We explored associations with burnout using multivariable logistic regression. We included clinically relevant covariates including age, gender, ethnicity, attending status, family medicine training background, pediatric training background, hospital type, number of shifts per month, awareness of emotional strategy use, number of emotional strategies used, and frequent use of each of the five emotional regulation strategies.

## RESULTS

Among 233 respondents who started the survey, 147 completed all questions (63.1%, Table 1). Of these, roughly half (53.1%) were aged 35–54 years, 54.4% were female, 20.4% identified as visible minorities and over a quarter (26.9%) were trainees. There was at least one participant from every province except for Prince Edward Island.

**TABLE 1:** Baseline characteristics stratified by burnout outcome.

Characteristics	Overall (*n* = 147)	Burnout on single‐item measures from the MBI (*n* = 66)	Burnout on the CBI (*n* = 65)
Age (years)			
<35	53 (36.1)	24 (36.4)	20 (30.8)
35–54	78 (53.1)	38 (57.6)	41 (63.1)
≥55	16 (10.9)	4 (6.1)	4 (6.2)
Gender			
Female	80 (54.4)	37 (56.1)	41 (63.1)
Male	66 (44.9)	28 (42.4)	23 (35.4)
Nonbinary	1 (0.7)	1 (1.5)	1 (1.5)
Ethnicity			
Indigenous	0 (0)	0 (0)	0 (0)
Visible minority	30 (20.4)	12 (18.2)	11 (16.9)
White	117 (79.6)	54 (81.8)	54 (83.1)
Province			
British Columbia	28 (19.0)	12 (18.2)	10 (15.4)
Alberta	40 (27.2)	21 (31.8)	18 (27.7)
Saskatchewan	5 (3.4)	2 (3.0)	2 (3.1)
Manitoba	2 (1.4)	1 (1.5)	1 (1.5)
Ontario	51 (34.7)	22 (33.3)	24 (36.9)
Quebec	11 (7.5)	5 (7.6)	3 (4.6)
Nova Scotia	5 (3.4)	1 (1.5)	3 (4.6)
Prince Edward Island	0 (0)	0 (0)	0 (0)
Newfoundland and Labrador	3 (2.0)	1 (1.5)	2 (3.1)
New Brunswick	2 (1.4)	1 (1.5)	2 (3.1)
Yukon	0 (0)	0 (0)	0 (0)
Nunavut	0 (0)	0 (0)	0 (0)
Northwest Territories	0 (0)	0 (0)	0 (0)
Responsibility			
PGY‐1	6 (4.1)	0 (0)	1 (1.5)
PGY‐2	8 (5.4)	7 (10.6)	5 (7.7)
PGY‐3	6 (4.1)	3 (4.5)	2 (3.1)
PGY‐4	7 (4.8)	3 (4.5)	2 (3.1)
PGY‐5	9 (6.1)	5 (7.6)	3 (4.6)
Fellow	2 (1.4)	0 (0)	0 (0)
Attending	109 (74.1)	48 (72.7)	52 (80.0)
Training background			
FRCPC EM	87 (59.2)	41 (62.1)	33 (50.8)
CCFP EM	48 (32.7)	20 (30.3)	24 (36.9)
CCFP	6 (4.1)	4 (6.1)	5 (7.7)
FRCPC PEDS	2 (1.4)	0 (0)	0 (0)
FRCPC PEM	4 (2.7)	1 (1.5)	3 (4.6)
Hospital type			
Tertiary	120 (81.6)	52 (78.8)	50 (76.9)
Urban	11 (7.5)	6 (9.1)	4 (6.2)
Suburban	9 (6.1)	4 (6.1)	6 (9.2)
Rural	7 (4.8)	4 (6.1)	5 (7.7)
Shifts per month	11.3 (±3.30)	11.2 (±3.24)	11.0 (±3.12)

*Note:* Data are reported as *n* (%) or mean (±SD).

Abbreviations: CBI, Copenhagen Burnout Inventory; CCFP, Certification in the College of Family Physicians; FRCPC, Fellow of the Royal College of Physicians of Canada; MBI, Maslach Burnout Inventory; PGY, postgraduate year.

Sixty‐six (44.9%) participants screened positive for burnout on at least one of the single‐item measures for emotional exhaustion and depersonalization, and 65 (44.2%) participants screened positive for burnout on the CBI. The internal consistency of the CBI was 0.90 (personal‐related 0.91, work‐related 0.90, client‐related 0.92).

Resetting was the most used emotional regulation strategy (57.8%) and increased vigilance was the least used (30.6%, Table 2). Cognitive reappraisal (38.1%), distancing (37.4%), and distraction (34.0%) were used fairly equally. Respondents used an average of two emotional regulation strategies. When asked how important emotional regulation strategies are for supporting their ability to practice emergency medicine, 74 (50.3%) thought that they were important, and 56 (38.1%) thought that they were very important. In contrast, only 58.5% were consciously aware that they were using emotional regulation strategies.

**TABLE 2:** Emotional regulation awareness, importance, and use by burnout outcome.

Characteristics	Overall (*n* = 147)	Burnout on single‐item measures from the MBI (*n* = 66)	Burnout on the CBI (*n* = 65)
Aware of emotional regulation strategy usage	86 (58.5)	43 (65.2)	40 (61.5)
Importance of emotional regulation strategy usage			
Very unimportant	8 (5.4)	4 (6.1)	4 (6.2)
Unimportant	2 (1.4)	0 (0)	0 (0)
Neutral	7 (4.8)	2 (3.0)	2 (3.1)
Important	74 (50.3)	33 (50.0)	36 (55.4)
Very important	56 (38.1)	27 (40.9)	23 (35.4)
Number of emotional regulation strategies frequently used			
0	26 (17.7)	3 (4.5)	7 (10.8)
1	34 (23.1)	11 (16.7)	11 (16.9)
2	33 (22.4)	14 (21.2)	14 (21.5)
3	30 (20.4)	20 (30.3)	18 (27.7)
4	19 (12.9)	13 (19.7)	12 (18.5)
5	5 (3.4)	5 (7.6)	3 (4.6)
Number of emotional regulation strategies frequently used–mean (SD)	1.98 (±1.41)	2.67 (±1.30)	2.40 (±1.38)
Distancing frequent use	55 (37.4)	41 (62.1)	38 (58.5)
Distraction frequent use	50 (34.0)	42 (63.6)	33 (50.8)
Reappraisal frequent use	56 (38.1)	27 (40.9)	25 (38.5)
Vigilance frequent use	45 (30.6)	29 (43.9)	26 (40.0)
Resetting frequent use	85 (57.8)	37 (56.1)	34 (52.3)

*Note:* Data are reported as *n* (%) or mean (±SD).

Abbreviations: CBI, Copenhagen Burnout Inventory; MBI, Maslach Burnout Inventory.

**FIGURE 1:**
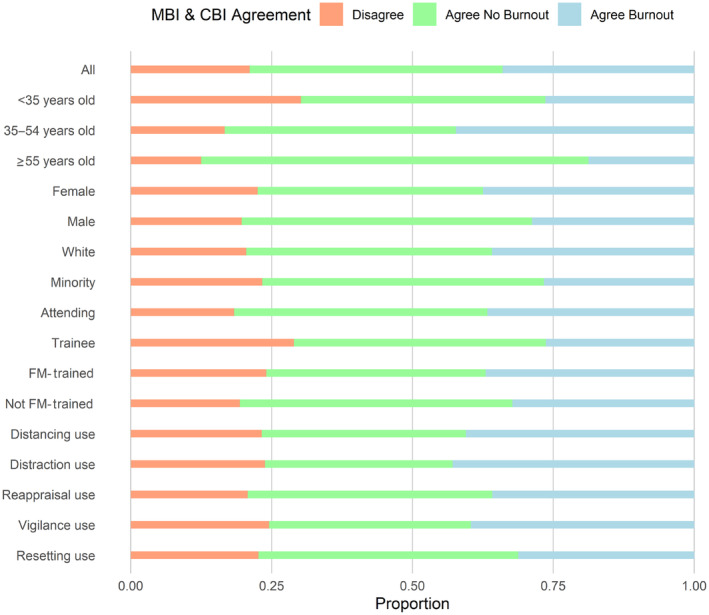
Agreement between Maslach Burnout Inventory (MBI) and Copenhagen Burnout Inventory (CBI).

The CBI and the single‐item measures from the MBI disagreed on 21.1% of all respondents (Figure 1). While this was fairly stable across tested subgroups, there were notable (non–statistically significant) differences among respondents from different age groups (30.2% for <35 years old vs. 12.5% for ≥55 years old, *p* = 0.12) as well as between trainees and attendings (28.9% vs. 18.3%, *p* = 0.25).

When controlling for other covariates (Table 3), frequent use of distraction had the strongest association with screening positive for burnout on the single‐item measures from the MBI (adjusted odds ratio [aOR] 14.4, 95% confidence interval [CI] [3.4–60.8]) and frequent use of distancing had the strongest association with screening positive on the CBI (aOR 10.1, 95% CI 2.5–39.8). Use of vigilance also had a strong association with burnout on the CBI (aOR 4.3, 95% CI 1.2–16.0). Visible minority status and awareness of emotional regulation strategy use were not associated with burnout.

**TABLE 3:** Association of physician characteristics with burnout.

	aOR (95% CI)	
Covariate	Burnout on single‐item measures from the MBI	Burnout on the CBI
35–54 years old	1.1 (0.2–4.6)	1.3 (0.4–4.5)
≥55 years old	0.3 (0.0–2.4)	0.3 (0.0–1.6)
Female	0.9 (0.4–2.3)	2.0 (0.9–4.5)
Visible minority	0.4 (0.1–1.5)	0.5 (0.2–1.5)
Attending	0.5 (0.1–2.8)	1.2 (0.2–5.3)
Pediatric training	0.0 (0.0–1.4)	3.3 (0.4–25.2)
FM training	0.3 (0.1–1.2)	1.2 (0.4–3.6)
Urban	2.8 (0.5–16.5)	0.4 (0.1–2.4)
Suburban	3.2 (0.3–30.6)	3.3 (0.5–24.1)
Rural	4.0 (0.5–32.3)	3.2 (0.4–24.6)
No. of shifts per month	0.9 (0.7–1.0)	0.9 (0.8–1.0)
Aware of emotional regulation strategies	1.7 (0.6–4.7)	1.2 (0.5–2.8)
Number of strategies used	1.1 (0.4–2.9)	0.6 (0.2–1.3)
Distancing use	3.7 (0.9–15.8)	10.1 (2.5–39.8)^a^
Distraction use	14.4 (3.4–60.8)^a^	2.7 (0.8–9.1)
Reappraisal use	0.2 (0.0–1.1)	1.0 (0.3–3.9)
Vigilance use	3.5 (0.9–14.1)	4.3 (1.2–16.0)^a^

*Note*: aOR for resetting use was not defined due to singularities.

Abbreviations: aOR, adjusted odds ratio; CBI, Copenhagen Burnout Inventory; FM, family medicine; MBI, Maslach Burnout Inventory.

^a^ Statistically significant.

## DISCUSSION

We sought to examine the conceptualization of burnout by comparing burnout scores from two validated measures. Despite near‐identical rates of burnout among respondents, the two measures disagreed over a fifth of the time, with noticeable (though not statistically significant) differences in agreement based on physician age and trainee status. We also found differences in which emotional regulation strategies were associated with burnout on the single‐item measures from the MBI as well as the CBI.

### Comparison to previous studies

Our study adds a novel comparison of two widely used burnout measures. Alahmari et al.^40^ previously reviewed the prevalence of burnout among healthcare workers and found that mean burnout was higher using the CBI (53%) than the MBI (35%) in the 38 studies they included. In contrast, Aleksa and Šertvytienė^41^ compared both inventories directly in a sample of Lithuanian medical professionals and found higher rates of severe burnout using the MBI (24.5% vs. 21.9%). We build on these studies by also examining agreement between these two instruments overall as well as among a few key subgroups. We are also the first to study and compare the above instruments among emergency physicians and residents.

Our results expand on Isbell et al.'s qualitative study,^22^ labeling their groupings of emotional regulation strategies and quantifying use among emergency physicians and trainees. The five emotional regulation strategies saw frequent use and were felt to be important in supporting participants' ability to practice emergency medicine, although nearly half were not consciously aware of their usage of emotional regulation strategies.

Frequent use of distraction and frequent use of distancing had the strongest association with screening positive for burnout on the single‐item measures from the MBI and the CBI, respectively. Previous reviews have documented associations between workload (shifts per month) and burnout.^42^, ^43^ They also show inconsistent results when examining age and gender,^42^, ^43^ although more recent studies in Canada have demonstrated higher rates of burnout in younger physicians and females.^1^, ^44^ In our study, none of these characteristics were associated with burnout after controlling for other confounders. While there was a trend toward lower burnout in older physicians, this was not statistically significant.

### Interpretation and implications

Evidently, the use of emotional regulation strategies is felt to be integral to help deal with the high‐stress, emotionally charged nature of emergency medicine practice. Despite this, a large proportion of practitioners are unaware of their active use of these strategies and suffer from burnout. Given that distancing, distraction, and vigilance use were all significantly associated with burnout, it is unclear whether individuals who feel burned out are more likely to use and benefit from these strategies or whether use of these strategies may lead to burnout long‐term. Further research is needed to elucidate this relationship and assess whether emotional regulation strategies are adaptive or maladaptive—or, perhaps, whether they are helpful in the short term (i.e., on shift) but contribute to the development of burnout in the long term.

When evaluating a measurement tool, construct validity refers to the degree to which a tool accurately represents the underlying construct. In this instance, the MBI and CBI have two different conceptualizations of the construct of burnout. The rate of disagreement and differences in association with emotional regulation strategies highlights that these conceptualizations are not equivalent from a practical point of view. As an emergency medicine community, we should therefore be careful when deciding which tool to use and how to interpret studies that use different burnout instruments. Although not reaching statistical significance, there was also a noticeably higher level of disagreement for respondents <35 years old and trainees, who largely overlap. Given that prior studies have shown higher rates of burnout in younger emergency physicians,^1^, ^44^ it is critical to further explore this phenomenon to ensure that burnout is being accurately assessed for these high‐risk groups.

A recent psychometric systematic review of burnout instruments did not make recommendations for one burnout instrument over another.^21^ Our study is not designed to draw conclusions of superiority or to make recommendations for a particular instrument; the choice of instrument should weigh the overall benefits and limitations of a tool. Evidently, there is a need to further study the validity and reliability of these burnout measures in diverse groups of emergency physicians, paying careful attention to consistent performance across important subgroups. Content validity in particular would be valuable to study to ensure that individual questions, the subscales, and the inventories as a whole are reflective of burnout as conceptualized by emergency physicians and trainees.

## LIMITATIONS

Although we recruited participants from across Canada using multiple channels, our study findings are limited in generalizability due to the small sample size. It is possible that individuals who are the most burned out may be less likely to complete the survey and therefore skew the overall prevalence of burnout lower. If the CBI and MBI are more consistent at detecting burnout in the most severe cases, nonresponse bias might also falsely raise the degree of disagreement observed in our study. However, a recent study found no differences in burnout between responders and nonresponders in a primary care setting.^45^ We were also unable to calculate nonresponse rates given the use of multimodal recruitment and the lack of information about how many potential participants saw the recruitment efforts. Furthermore, there were few pediatric emergency physicians, family medicine–trained providers, and rural physicians. It is also unclear whether these findings would be directly generalizable to other settings such as the United States where training and practice differ from that in Canada.

In addition to limitations relating to recruitment, the single‐item measures from the MBI are not a perfect representation of the full inventory. Therefore, comparing the full MBI with the CBI may have yielded more consistent agreement. However, despite this limitation, the single‐item measures from the MBI are one of the most commonly used burnout tools in physicians; therefore, we felt that it would be a helpful scale to compare with the CBI while minimizing time associated with completion of our survey. Only 63% of participants initiating the survey completed all questions; survey fatigue may have therefore affected the accuracy of questions in the latter part of the survey.

## CONCLUSIONS

While rates of burnout among emergency physicians and trainees are similar when using the Copenhagen Burnout Inventory and single‐item measures of emotional exhaustion and depersonalization from the Maslach Burnout Inventory, agreement between the two measures varies, and there is a trend toward increased discrepancy among trainees and younger emergency physicians. Future research is needed to elucidate this relationship as well as to examine the validity and reliability of burnout measures in this population. While the use of distancing, distraction, and vigilance were strongly associated with burnout, most physicians did so subconsciously and felt they were very important in supporting their ability to practice emergency medicine. We must further explore whether emotional regulation strategies are adaptive or maladaptive to better identify how to improve emergency physicians' well‐being.

## AUTHOR CONTRIBUTIONS

Henry Li conceptualized the study. All authors contributed to the study design. Henry Li and Leandro Solis Aguilar contributed to the acquisition and analysis of the data. All authors contributed to the interpretation of the data. Henry Li drafted the manuscript, and the remaining authors reviewed it critically for important intellectual content.

## FUNDING INFORMATION

This work was supported by the Edmonton Emergency Physicians Association and the Professional Association of Resident Physicians of Alberta.

## CONFLICT OF INTEREST STATEMENT

The authors have no conflicts of interest to declare.

## APPENDIX A Checklist for reporting of survey studies

**Table:**

Section/topic	Item	Item description	Reported on page #
Title and abstract			
Title and abstract	1a	State the word “survey” along with a commonly used term in title or abstract to introduce the study's design	1
	1b	Provide an informative summary in the abstract, covering background, objectives, methods, findings/results, interpretation/discussion, and conclusions	1–2
Introduction			
Background	2	Provide a background about the rationale of study, what has been previously done, and why this survey is needed	3–4
Purpose/aim	3	Identify specific purposes, aims, goals, or objectives of the study	4
Methods			
Study design	4	Specify the study design in the methods section with a commonly used term (e.g., cross‐sectional or longitudinal)	4
	5a	Describe the questionnaire (e.g., number of sections, number of questions, number and names of instruments used)	6
Data collection methods	5b	Describe all questionnaire instruments that were used in the survey to measure particular concepts. Report target population, reported validity and reliability information, scoring/classification procedure, and reference links (if any)	6‐7
	5c	Provide information on pretesting of the questionnaire, if performed (in the article or in an online supplement). Report the method of pretesting, number of times questionnaire was pre‐tested, number and demographics of participants used for pretesting, and the level of similarity of demographics between pre‐testing participants and sample population	6‐7
	5d	Questionnaire if possible, should be fully provided (in the article, or as appendices or as an online supplement)	Appendix A
Sample characteristics	6a	Describe the study population (i.e., background, locations, eligibility criteria for participant inclusion in survey, exclusion criteria)	5
	6b	Describe the sampling techniques used (e.g., single stage or multistage sampling, simple random sampling, stratified sampling, cluster sampling, convenience sampling). Specify the locations of sample participants whenever clustered sampling was applied	7
	6c	Provide information on sample size, along with details of sample size calculation	7
	6d	Describe how representative the sample is of the study population (or target population if possible), particularly for population‐based surveys	7
Survey administration	7a	Provide information on modes of questionnaire administration, including the type and number of contacts, the location where the survey was conducted (e.g., outpatient room or by use of online tools, such as SurveyMonkey)	7
	7b	Provide information of survey's time frame, such as periods of recruitment, exposure, and follow‐up days	7
	7c	Provide information on the entry process: –>For non‐web‐based surveys, provide approaches to minimize human error in data entry –>For web‐based surveys, provide approaches to prevent “multiple participation” of participants	N/A
Study preparation	8	Describe any preparation process before conducting the survey (e.g., interviewers’ training process, advertising the survey)	6
Ethical considerations	9a	Provide information on ethical approval for the survey if obtained, including informed consent, institutional review board [IRB] approval, Helsinki declaration, and good clinical practice [GCP] declaration (as appropriate)	5
	9b	Provide information about survey anonymity and confidentiality and describe what mechanisms were used to protect unauthorized access	5
Statistical analysis	10a	Describe statistical methods and analytical approach. Report the statistical software that was used for data analysis	7‐8
	10b	Report any modification of variables used in the analysis, along with reference (if available)	N/A
	10c	Report details about how missing data was handled. Include rate of missing items, missing data mechanism (i.e., missing completely at random [MCAR], missing at random [MAR] or missing not at random [MNAR]) and methods used to deal with missing data (e.g., multiple imputation)	8
	10d	State how non‐response error was addressed	N/A
	10e	For longitudinal surveys, state how loss to follow‐up was addressed	N/A
	10f	Indicate whether any methods such as weighting of items or propensity scores have been used to adjust for non‐representativeness of the sample	N/A
	10g	Describe any sensitivity analysis conducted	N/A
Results			
Respondent characteristics	11a	Report numbers of individuals at each stage of the study. Consider using a flow diagram, if possible	8
	11b	Provide reasons for non‐participation at each stage, if possible	N/A
	11c	Report response rate, present the definition of response rate or the formula used to calculate response rate	N/A
	11d	Provide information to define how unique visitors are determined. Report number of unique visitors along with relevant proportions (e.g., view proportion, participation proportion, completion proportion)	N/A
Descriptive results	12	Provide characteristics of study participants, as well as information on potential confounders and assessed outcomes.	8, Table 1
Main findings	13a	Give unadjusted estimates and, if applicable, confounder‐adjusted estimates along with 95% confidence intervals and *p*‐values	8–9, Table 3
	13b	For multivariable analysis, provide information on the model building process, model fit statistics, and model assumptions (as appropriate)	7–8
	13c	Provide details about any sensitivity analysis performed. If there are considerable amount of missing data, report sensitivity analyses comparing the results of complete cases with that of the imputed dataset (if possible)	N/A
Discussion			
Limitations	14	Discuss the limitations of the study, considering sources of potential biases and imprecisions, such as non‐representativeness of sample, study design, important uncontrolled confounders	12–13
Interpretations	15	Give a cautious overall interpretation of results, based on potential biases and imprecisions and suggest areas for future research	11–12
Generalizability	16	Discuss the external validity of the results	13
Other sections			
Role of funding source	17	State whether any funding organization has had any roles in the survey's design, implementation, and analysis	Title page
Conflict of interest	18	Declare any potential conflict of interest	Title page
Acknowledgements	19	Provide names of organizations/persons that are acknowledged along with their contribution to the research	Title page

## APPENDIX B

(A) Demographics

1. Are you an emergency physician or resident practicing in Canada?
  - Yes
  - No
2. In which province or territory do you work the majority of your emergency department shifts? (Select all that apply)
  - British Columbia
  - Alberta
  - Saskatchewan
  - Manitoba
  - Ontario
  - Quebec
  - Nova Scotia
  - PEI
  - Newfoundland and Labrador
  - New Brunswick
  - Yukon
  - Nunavut
  - Northwest Territories
3. What best describes the emergency department in which you work in most of the time? (Select all that apply)
  - Tertiary care centre
  - Urban hospital (not tertiary care)
  - Suburban hospital
  - Rural or remote hospital
4. How many shifts do you work in a typical month while working in the emergency department?
  - Drop down 1–30
5. Please indicate your age
  - <35
  - 35–54
  - 55+
6. Please indicate the gender you identify with
  - Female
  - Male
  - Non‐binary
  - Prefer not to answer
7. The Government of Canada's Canada Employment Equity Act and Statistics Canada both define visible minorities as persons—other than Aboriginal peoples—who are non‐white in color. General groupings defined by Statistics Canada for the visible minority variable are included below. We recognize that there may be a preference to instead identify as a “person of color,” or by an individual's race or ethnicity. However, for the purposes of this question, please use the definition provided by the Canada Employment Equity Act and Statistics Canada. Do you identify as:
  - Indigenous/Aboriginal
  - Visible Minority
    - Visible minority refers to whether a person is a visible minority or not, as defined by the Employment Equity Act. The Employment Equity Act defines visible minorities as &quot;persons, other than Aboriginal peoples, who are non‐Caucasian in race or non‐white in color&quot. The visible minority population consists mainly of the following groups: South Asian, Chinese, Black, Filipino, Arab, Latin American, Southeast Asian, West Asian, Korean and Japanese.
  - White
  - Prefer not to answer
8. What is the highest level of responsibility you currently have? For example, fellows who concurrently work as an attending in an emergency department should select “Attending”
  - PGY1
  - PGY2
  - PGY3
  - PGY4
  - PGY5
  - Fellow
  - Attending
9. What is your training background/program? (Select all that apply)
  - FRCPC in Emergency Medicine
  - CCFP‐EM
  - CCFP
  - FRCPC in General Pediatrics
  - PEM Fellowship
  - Other (please specify)
10. If you are an attending, how many years have you been in practice?
  - ≤5
  - 6‐10
  - 11–20
  - 21–30
  - >30

(B) Maslach Burnout Inventory & Emotional Regulation Strategies

11 Please indicate how often you've felt the following as it relates to your job:
  - I feel burned out from my work
    - Never
    - A few times a year or less
    - Once a month or less
    - A few times a month
    - Once a week
    - A few times a week
    - Every day
  - I have become more callous toward people since I took this job
    - Never
    - A few times a year or less
    - Once a month or less
    - A few times a month
    - Once a week
    - A few times a week
    - Every day
12 How often do you use the following strategies to manage negative emotions on shift in the emergency department? You may or may not be aware of using these strategies. Please thoughtfully consider whether these occur.
  - Emotional distancing (e.g. actively suppressing or disengaging from emotions during and/or after interactions, compartmentalizing interactions and associated emotions)
    - Never
    - Rarely
    - Some shifts
    - Most shifts
    - Every shift
  - Distraction (e.g. redirecting focus/attention away from your emotions and toward task‐oriented activity such as the volume and/or acuity of tasks)
    - Never
    - Rarely
    - Some shifts
    - Most shifts
    - Every shift
  - Cognitive reappraisal (e.g. intentionally revising your initial subjective interpretation of an event, interaction or environment into a more positive light such as choosing to reframe a challenging interaction with a patient as their attempt to express an unmet need)
    - Never
    - Rarely
    - Some shifts
    - Most shifts
    - Every shift
  - Increased vigilance (e.g. reflecting on emotions that may arise prior to approaching a potentially challenging situation, with consideration for the impact of these emotions)
    - Never
    - Rarely
    - Some shifts
    - Most shifts
    - Every shift
  - Resetting away from patients (e.g. taking breaks, speaking with colleagues, using humor, or engaging in self‐care behaviors, to offset stress and help manage the emotional burden)
    - Never
    - Rarely
    - Some shifts
    - Most shifts
    - Every shift
13 Were you consciously aware that you used emotional regulation strategies (such as those listed above or others) prior to responding to these questions?
  - Yes
  - No
14 What other strategies do you use to regulate your emotions relating to your work in the emergency department?
  - Open‐ended
15 How important do you think emotional regulation strategies are in supporting your ability to practice emergency medicine?
  - Very unimportant
  - Unimportant
  - Neutral
  - Important
  - Very important
16 What additional comments or clarifications do you have relating to emotional regulation strategies in the emergency department?
  - Open‐ended

(C) Copenhagen Burnout Inventory

17 Do you feel worn out at the end of the working day?
  - Always
  - Often
  - Sometimes
  - Seldom
  - Never/almost never
18 Are you tired of working with patients?
  - Always
  - Often
  - Sometimes
  - Seldom
  - Never/almost never
19 Are you exhausted in the morning at the thought of another day at work?
  - Always
  - Often
  - Sometimes
  - Seldom
  - Never/almost never
20 Do you sometimes wonder how long you will be able to continue working with patients?
  - Always
  - Often
  - Sometimes
  - Seldom
  - Never/almost never
21 How often do you feel tired?
  - Always
  - Often
  - Sometimes
  - Seldom
  - Never/almost never
22 Do you feel that every working hour is tiring for you?
  - Always
  - Often
  - Sometimes
  - Seldom
  - Never/almost never
23 Do you find it frustrating to work with patients?
  - To a very high degree
  - To a high degree
  - Somewhat
  - To a low degree
  - To a very low degree
24 Do you feel that you give more than you get back when you work with patients?
  - To a very high degree
  - To a high degree
  - Somewhat
  - To a low degree
  - To a very low degree
25 How often do you feel worn out?
  - Always
  - Often
  - Sometimes
  - Seldom
  - Never/almost never
26 Do you find it hard to work with patients?
  - To a very high degree
  - To a high degree
  - Somewhat
  - To a low degree
  - To a very low degree
27 How often do you feel weak and susceptible to illness?
  - Always
  - Often
  - Sometimes
  - Seldom
  - Never/almost never
28 Is your work emotionally exhausting?
  - To a very high degree
  - To a high degree
  - Somewhat
  - To a low degree
  - To a very low degree
29 Does your work frustrate you?
  - To a very high degree
  - To a high degree
  - Somewhat
  - To a low degree
  - To a very low degree
30 How often are you physically exhausted?
  - Always
  - Often
  - Sometimes
  - Seldom
  - Never/almost never
31 How often do you think: “I can't take it anymore”?
  - Always
  - Often
  - Sometimes
  - Seldom
  - Never/almost never
32 Do you feel burned out because of your work?
  - To a very high degree
  - To a high degree
  - Somewhat
  - To a low degree
  - To a very low degree
33 Does it drain your energy to work with patients?
  - To a very high degree
  - To a high degree
  - Somewhat
  - To a low degree
  - To a very low degree
34 Do you have enough energy for family and friends during leisure time?
  - Always
  - Often
  - Sometimes
  - Seldom
  - Never/almost never
35 How often are you emotionally exhausted?
  - Always
  - Often
  - Sometimes
  - Seldom
  - Never/almost never
36 Do you have any other thoughts about emotional regulation strategies and the measurement of burnout in emergency physicians?
  - Open‐ended

If you are experiencing mental health concerns or psychological distress, there are a variety of resources available to help:

Provincial/Territorial Physician Health Programs: https://www.cma.ca/support.

Talk Suicide Canada (National Crisis Line): 1‐833‐456‐4566 (24/7), or by text message at 45645.

Hope for Wellness Helpline (Culturally sensitive counseling for Indigenous Peoples): 1‐855‐242‐3310.

Tracom Crisis Intervention (Bilingual): 514‐483‐3033 (24/7).
